# Alzheimer’s Disease Diagnosis by Detecting Exogenous Fluorescent Signal of Ligand Bound to Beta Amyloid in the Lens of Human Eye: An Exploratory Study

**DOI:** 10.3389/fneur.2013.00062

**Published:** 2013-05-27

**Authors:** Charles Kerbage, Carl H. Sadowsky, Danna Jennings, Gerald D. Cagle, Paul D. Hartung

**Affiliations:** ^1^Cognoptix, Inc., Acton, MA, USA; ^2^Premiere Research Institute, Nova Southeastern University, West Palm Beach, FL, USA; ^3^Molecular NeuroImaging LLC, New Haven, CT, USA

**Keywords:** Alzheimer’s disease, diagnosis, human eyes, beta amyloid, biomarker

## Abstract

We report results of a clinical exploratory human trial involving 10 participants using a combination of a fluorescent ligand and a laser scanning device, SAPPHIRE System, as an aid in the diagnosis of Probable Alzheimer’s disease (AD). To the best of our knowledge, this is the first time that such a technique has been used *in vivo* of a human lens. The primary goal of the clinical trial, in addition to safety assessment, was to evaluate efficacy of the system. By detecting specific fluorescent signature of ligand bound beta amyloid in the supranucleus (SN) region of the human lens, a twofold differentiation factor between AD patients and Control groups is achieved. Data from our studies indicates that deeper regions of the SN provide the highest measures of ligand bound fluorescence signal from both controls and patients with AD. In addition, we present preclinical studies that were performed to investigate the binding affinity of the ligand to beta amyloid and evaluate the pharmacokinetics of the ligand in rabbit eyes. Further studies are underway involving a larger population for statistical evaluation of the method.

## Introduction

Alzheimer’s disease (AD) is the leading neurodegenerative disorder in the world today and accounts for approximately two-thirds of cases of dementia. Globally, it affects around 10% of people above the age of 65. In the United States alone, approximately five million people suffer from AD-related dementia (Brookmeyer et al., 2011) with annual associated costs estimated in the range of $183 billion (Alzheimer’s Association, 2011). Definite diagnosis of AD is performed post mortem through histopathological identification of characteristic features including beta-amyloid plaques. Currently, AD is a clinical diagnosis based on NINCDS-ADRDA and DSM-IV criteria (McKhann et al., 1984, 2011; Hyman et al., 2012). The major features are late onset progressive impairment of short term memory associated with deterioration of language and visuospatial functions in the absence of disturbance of consciousness and systemic disorders (Esteban-Santillan et al., 1998; Erkinjuntti and Rockwood, 2003).

According to the amyloid cascade hypothesis, the accumulation of β-amyloid (Aβ) deposits as amyloid plaques in the patient’s brain is the primary event in the pathogenesis of AD (Hardy and Selkoe, 2002; Jack et al., 2010; Karran et al., 2011; Reiman et al., 2012). A post mortem diagnosis of AD is based on the presence of extracellular neuritic plaques positive for Aβ protein, dystrophic neurites, and intracellular NFTs in the brain. Amyloidal plaques are composed of 40–42 amino acid Aβ-folded peptides (McKhann et al., 2011; Hyman et al., 2012). Incidental AD-like pathology can be found in cognitively normal individuals over the age of 75, but large deposits of Aβ suggest active AD (Hardy and Selkoe, 2002). In addition, recent studies demonstrated that functional and structural brain imaging changes are consistent with Aβ1–42 overproduction, in young adult PSEN1E280A mutation carriers (Karran et al., 2011; Reiman et al., 2012). Draft guidelines issued in 2011 by the Alzheimer’s Association and NIA (Alzheimer’s Association, 2011) recommend the incorporation of Aβ biomarkers in the diagnosis of AD for research purposes.

Presently, there is a major effort investigating the possibility of diagnosing AD by detecting the presence and accumulation of protein Aβ. Technologies such as positron emission tomography (PET) imaging (Klunk et al., 2004; Nordberg, 2004; Doraiswamy et al., 2012), magnetic resonance (MR) microscopy (Hintersteiner et al., 2005), and optical imaging (Yang et al., 2012) have been demonstrated as potential tools to enable detection of amyloid plaques in living AD patients and animal models. In particular, florbetapir, which is a radioactive tracer, was recently approved by the U.S. Food and Drug Administration for PET imaging of the brain to estimate beta-amyloid plaque density in patients who are being evaluated for cognitive impairment (Nakada et al., 2008). However, most of these techniques have some limitations in resolution and are costly. There is a clear need for a technology capable of improving the diagnosis of AD with high resolution sensitivity and specificity that offers ease of use during the testing and that will reasonably priced.

Current studies have been focused on detecting beta-amyloid in the eye as a technique to aid in the diagnosis of AD in humans (Goldstein et al., 2003; Moncaster et al., 2010; Koronyo-Hamaoui et al., 2011) and animal models (Koronyo et al., 2012; Parnell et al., 2012). Frederikse (2000) showed lens protein β-sheet arrays are organized in an amyloid protein supramolecular order in the interior fiber cells of mammalian ocular lens. Also, Goldstein et al. (2003) demonstrated the presence of Aβ deposits in the supranucleus (SN) of the lens of the eye in a population in whom AD had been confirmed by autopsy and differentiated from control samples. Further studies showing similar results have been extended to subjects with Down syndrome (Moncaster et al., 2010).

In this paper, we report the results of a clinical exploratory study involving 10 participants, 5 AD patients and 5 Control, performed with SAPPHIRE System, which is a combination of a fluorescent ligand, named Compound #11, and a laser scanning device, to aid in the diagnosis of probable AD in patients by detecting the presence of beta-amyloid in the eye of human beings who have symptoms and signs consistent with Alzheimer’s type dementia. Measurements were performed on three different locations in the SN of the eye and show that deeper regions (thickness of 900 μm) show higher level of ligand bound to Aβ. Further clinical studies with a larger population are being performed for statistical validation of the system.

## Materials and Methods

### Fluorescent ligand

**Figure 1:**
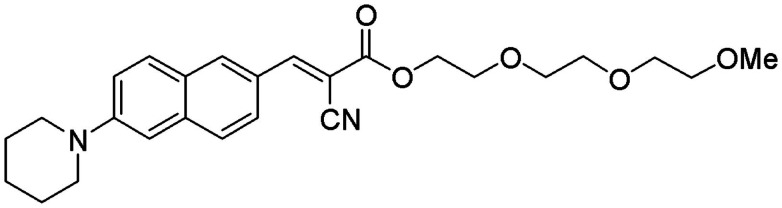
**Chemical structure of Compound #11**.

Compound #11 is a new fluorescent compound that was designed according to the molecular rotor motif, which has been shown to bind to the aggregated Aβ peptide (Sutharsan et al., 2010) (Figure 1).

Compound #11, whose chemical name is (E)-2-[2-(2-methoxyethoxy)ethoxy]ethyl-2-cyano-3-[6-(piperidin-1-yl)naphthalene-2-yl]acrylate is formulated at 0.5% into an ophthalmic Ointment consisting of 80% petrolatum and 19.5% mineral oil for topical application.

**Figure 2:**
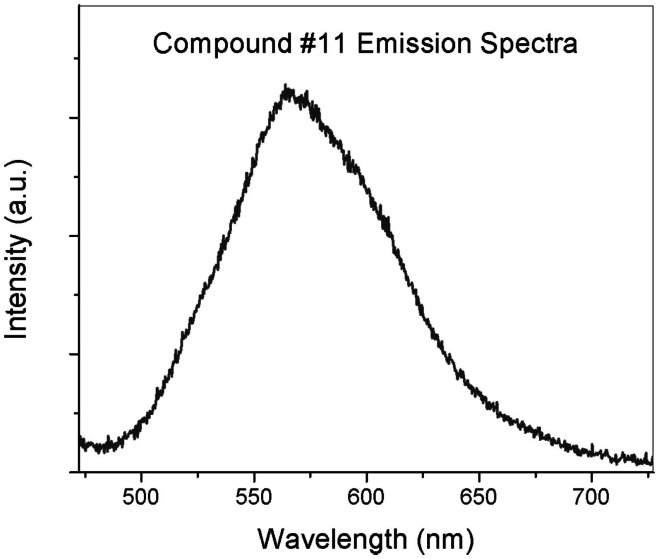
**Emission spectrum (585 nm) of Compound #11 (excitation 470 nm)**.

Fluorescence characterization of the ligand in buffer solution shows a large Stokes shift, the difference between the emission and the excitation peaks, of ∼90 nm and FWHF of 80 nm when excited at 470 nm (Figure 2).

### Ophthalmic ointment preparation

The Ophthalmic Ointment was manufactured by Bio-Concept Laboratories, Salem, NH in compliance with the current good manufacturing practices (cGMP) as set forth in the Code of Federal Regulations, Section 21 parts 210 and 211. The suspension was first compounded, filled into 1 mL syringes, sterilized by e-beam irradiation, and then sealed in foil pouched.

### Laser scanning device

The laser scanning device is designed to measure emitted fluorescence signal of Compound #11 bound to Aβ aggregates in the supranuclear region of the lens, the region of interest (ROI). The device comprises of fluorescence lifetime technique that is based on short pulse excitation repetitively and recording the subsequent fluorescence light emission as a function of time. In particular, time domain fluorescence lifetime spectroscopy has been demonstrated as a powerful tool to enable separation of fluorophores based on their lifetime signatures (Lakowicz, 2006; Koberling et al., 2007). In our application, the fluorescence lifetime decay rate is registered for each location scanned in the human lens and thus an extra dimension of the fluorophores distributions can be evaluated and analyzed based on both fluorescence decay rate and the increase in fluorescence intensity upon binding to Aβ aggregates as mentioned earlier.

The optical scanner device itself comprises of a pico-second pulsed laser (PicoQuant, Berlin) with a peak wavelength at 470 nm, pulse width 200 ps, 40 MHz repetition rate, and average output power of 4 μW. Fluorescence from excited molecules is collected in epi-fluorescence configuration, filtered with dichroic mirrors (Semrock Inc.) and an additional bandpass filter (centered at 585 nm) to reject remaining scattered laser light, and passed through an aperture to enable confocal detection. The detector is a single photon avalanche diode (MPD, Bolzano, Italy) with 50 ps FWHM timing resolution and efficiency of 50% at 550 nm.

### *In vitro* characterization

#### Aggregated Aβ peptides preparation

Aggregated Aβ peptide was prepared by dissolving Aβ(1-42) in PBS pH 7.4 to a final concentration of 100 μM. This solution was magnetically stirred at 1200 rpm for 3 days at room temperature. The 100 μM Aβ(1–42) stock solution in PBS was aliquoted and frozen at −80 °C for up to 4 weeks without noticeable change in its property. One hundred fifty microliters of pre-aggregated Aβ(1–42) was added to 2.85 mL of Compound #11 to attain a concentration of 5 μM Aβ(1–42) and 4 μM of Compound #11. The solution was then transferred to 5 mL vial with buffer solution and the fluorescence lifetime measurements were performed at room temperature.

#### *In vitro* binding properties to amyloid plaques

**Figure 3:**
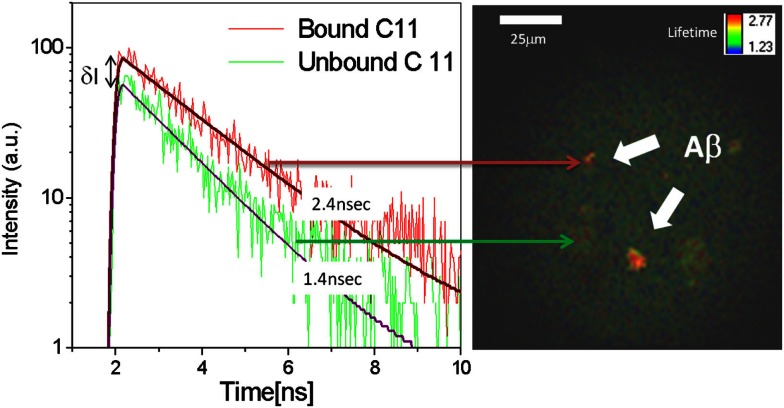
**Fluorescence decay rates for bound (red) and unbound (green) Compound #11 to Aβ peptide**. Increase in fluorescence intensity (δI ∼ 2X) and decay rates of bound (τ = 2.4 ns) Compound #11 vs. unbound (τ = 1.4 ns) to Aβ. Fluorescence lifetime image of aggregated Aβ peptide in color coded representation.

*In vitro* experiments were conducted to characterize the binding signal of beta-amyloid to Compound #11. The sample described above was scanned with the device and fluorescence lifetime image is obtained and shown in Figure 3 along with a plot of fluorescence decay rates of unbound (green) and bound (red) Compound #11 to aggregated Aβ peptide. The *in vitro* fluorescence lifetime measurements of Compound #11 with aggregated Aβ peptide demonstrate that bound and unbound peptide to Compound #11 can be resolved and differentiated with 0.85 ns difference in lifetime and at a detection level of few 100 photons. Thus, by combining fluorescence intensity and lifetime measurements, additional information is obtained to discriminate among several fluorescent labels and more importantly between bound and unbound ligand and differentiate that from natural fluorescence in the lens of the eye.

### Quantitative analysis

By knowing the signature of the fluorescent ligand, the lifetime decay histogram can be decomposed based on multiple exponential fittings and the frequency counts of photons with the specific decay lifetime (i-photons/s) are calculated. Since the fluorescence decay time is a characteristic for each fluorescence molecule, one can determine and separate the ligand from natural fluorescence of the lens that is being excited in the sample volume. In multi-exponential model, which is the case of mixture of fluorophores, the intensity is assumed to decay as the sum of individual single exponential decays: $I\left(t\right)=\sum_{i=1}^{n}\alpha_{i}\times e^{t∕\tau_{i}}+\beta$; where τ*_i_* is the decay time, α*_i_* is the amplitude of the components at *t* = 0, and *n* is the number of decay times. The values of α*_i_* and τ*_i_* can be used to determine the fractional contribution (*i_p_*) of each decay time. The situation where one does not expect a limited number of discrete decay times, rather a distribution of decay times, such behavior is expected for a mixture of fluorophores. Therefore, the intensity decays are typically analyzed in terms of a lifetime distribution. Thus, the α*_i_* values are replaced by distribution function (α_τ_) and the component with individual τ value is given by *I*(τ, *t*) = α(τ)*e*^−*t*/τ^. However, one cannot observe these individual components with their specific lifetime τ, but only the entire decay and the total decay is the sum of the individual decays weighted amplitudes. By analogy with the multi-exponential model, it is possible to rewrite the amplitude associated with the *i*th component of distribution as $i-\text{photons = }\frac{\int_{0}^{n}\alpha_{i}\left(\tau \right)\tau d\tau }{\int_{0}^{n}\sum_{i}\alpha_{i}\left(\tau \right)\tau d\tau }$

### Animal studies for safety assessments

Animal studies were performed with the device and the ligand [Compound #11 (0.5%), Ophthalmic Ointment] for acute toxicity and photo toxicity on Dutch-belted rabbits. During the course of the study, animals were dosed 12 times with Compound #11 Ointment (0.5%) over 4 days. Animals were examined with the device at the beginning and end of each day. Ocular examinations of animals treated with Compound #11 Ointment, did not present any significant signs of ocular toxicity. Additionally, the test article did not exhibit signs of phototoxicity.

### Animal studies for pharmacokinetics assessments

**Figure 4:**
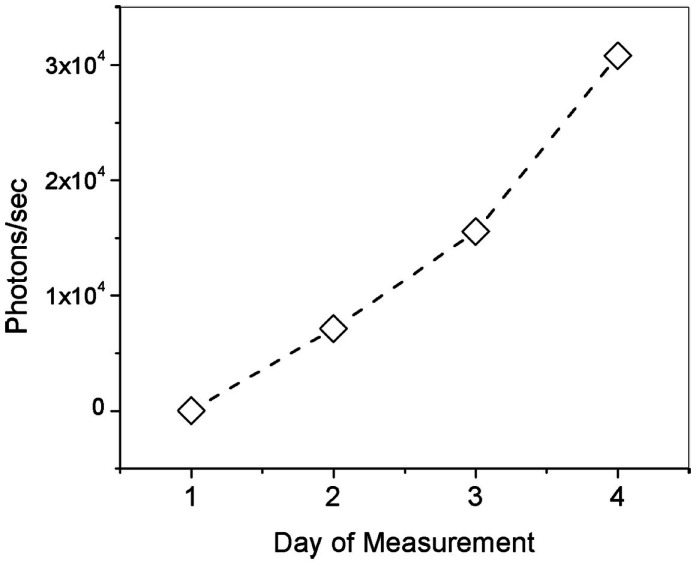
**Number of photons/s indicative of unbound Compound #11 ligand (τ = 1.4 ns) in the lens of rabbit eyes along a 4-day pharmacokinetics study**.

Ocular pharmacokinetic investigation of dose response via topical administration of fluorescence ligand was performed in Dutch-belted rabbits. The animals were anesthetized with subcutaneous injection of Dexdomitor (0.5 mg/kg), Ketamine (5 mg/kg). A ribbon of Ointment approximately 1/2″ long was then applied to the lower right eyelid of each animal in the test group three times a day for 4 days. Figure 4 is a plot showing the number of photon/sec indicative of Compound #11 measured in the lens nucleus of the rabbit eye every morning after being dosed during the 4-day study period. The measurements on the rabbits, which were dosed with Compound #11 Ophthalmic Ointment, show significant increase in fluorescence signal indicative of the ligand in the nucleus of the eye along the 4-day study period. Figure 4 is a plot of number of photons indicative of unbound ligand and shows that after three dosages each day with 3 h separation, cumulative fluorescence signal is detected and represent a total amount of 280 ng/mL of ligand.

### Study design and participants

The primary objective of this study was to determine the safety of the combination system of device and fluorescent ligand. The study was an exploratory open-label, parallel group study of five AD subjects and five healthy volunteers, Control Group, requiring four visits.

The study was conducted in accordance with the current revision of Declaration of Helsinki guiding physicians in medical research involving human subjects. The New England Institutional Review Board, Newton, MA, USA, approved the protocol to initiate the clinical study.

A “time and events” schedule for the study is summarized in Table A1 in Appendix. Visit 1 was a screening visit which included several procedures with an overall objective of ensuring each individual met the inclusion/exclusion criteria for participation in the clinical study. Importantly, an eye exam and neurological/psychometric evaluations were performed to qualify potential participants against inclusion/exclusion criteria. Visit 2 included baseline (pre-dose) measurements with the SAPPHIRE device, and the first three doses with the Compound #11 Ointment. Visit 3 included fluorescence measurements with the SAPPHIRE device to assess the effect of the first three doses with the Compound #11 Ointment, administration of three additional doses and fluorescence scanning measurements 4 h after the last dose. A full safety exam including an ophthalmic exam, physical exam, and blood work were performed at the end of the day. It is important to mention that the additional three doses during this visit did not show any substantial changes in the results from the first three doses in terms of safety or fluorescence measurements. Visit 4 was a 7-day follow-up safety exam only.

Adverse events (AEs) and vital signs were monitored at each application of ligand and at each measurement session with the device.

### Inclusion criteria

Subjects met the following inclusion criteria to participate in the study. A Clinical Dementia Rating (CDR) score of 1 or 2 for AD and 0 for Control group. All subjects in this study must have had at least one intact ocular lens that has not been exposed to surgery, trauma, or glaucoma. All subjects in both cohorts were high school graduates.

#### AD subjects

Alzheimer’s disease subjects were >50 years of age, male or female. All AD subjects met NINCDS-ADRDA criteria for Probable AD and DSM-IV criteria for Dementia of the Alzheimer’s Type. AD subjects did not fulfill the ICC criteria for probable DLB, the NINDS-AIREN for probable VaD, or the Neary criteria for FTD. All AD subjects received an MRI. In each case, the brain scans were negative for changes associated with stroke and/or generalized cerebrovascular disease (e.g., the ARWMC scale). Changes observed from MRI scans were limited to a white matter lesion score of 0 or 1 or 2 and a basal ganglia score of 0 or 1. All subjects in this group were high school graduates. The Protocol required that women of childbearing potential receive pregnancy testing before entry into the clinical trial. Of the five probable AD patients, two were female. Both were post-menopausal, ages were 65 and 73.

#### Control group

The Protocol required that Control group subjects be >20 years of age. Five normal males were enrolled in Study NEU02, age 31–77. All healthy volunteers had no evidence of cognitive impairment and had a Mini-Mental State Exam (MMSE) score between 28 and 30. Control group had Consortium to Establish a Registry for Alzheimer’s Disease (CERAD) neuropsychological test battery *z*-score of ≥(−1.00) for each subtest (except the MMSE which is covered by the criteria above). Additionally, HVs had an MRI brain scan that was judged as “normal (age appropriate)” including ARWMC scale scores supporting the lack of cerebrovascular disease (e.g., a white matter lesion score of 0 or 1 or 2 and a basal ganglia score of 0 or 1) and a Sheltens scale verifying the lack of cerebral atrophy (e.g., bilateral temporal lobe atrophy visual score of 0 or 1). All subjects in this group of volunteers were high school graduates.

#### Exclusion criteria

- History of learning or developmental disability
- History of serious active cardiac condition or significant small vessel disease (e.g., confluent white matter alterations on structural MRI or computed tomography scan)
- History of other neurologic condition (e.g., large vessel stroke, seizures, Parkinsonism)
- History of cataract surgery in the test eye
- History of physical injury to the test eye
- Retinal detachment
- Any lens opacity that prevents the operator from visualizing the lens sufficiently to perform the procedure
- Pregnancy
- Nursing women
- Systemic illness that may pose an unacceptable risk to the subject in the judgment of the investigator

##### Participants

Five AD subjects (two females, three males) ranging from 65 to 83 years old met the NINCDS-ADRDA criteria for Probable AD and DSM-IV criteria for Dementia of the Alzheimer’s Type set by the CERAD. Table 1 is a list of the AD subjects recruited for the study showing the age, sex, and test scores. The AD subjects all had a score of 1 on the CDR scale. Also, the AD subjects had MRI brain scan findings that do not reveal changes indicative of stroke and/or generalized cerebrovascular disease; changes limited to: a white matter lesion score of 0 or 1 or 2 and a basal ganglia score of 0 or 1.

**Table 1:** **AD subjects recruited for the study**.

Subject	Age	MMSE	CDR	Total CERAD	Sex
0004	65	23	1	93	F
2001	68	22	1	58	M
2002	77	23	1	53	M
2004	83	24	1	61	M
2007	73	22	1	54	F

Control subjects (five males) with ages 31–77 years old were recruited and had no evidence of cognitive impairment with a MMSE score between 28 and 30 (Table 2). The Control subjects all had a CDR score of 0. Additionally, controls had an MRI brain scan that has been judged as “normal (age appropriate)” including ARWMC scale scores supporting the lack of cerebrovascular disease (e.g., a white matter lesion score of 0 or 1 or 2 and a basal ganglia score of 0 or 1) and a Sheltens scale verifying the lack of cerebral atrophy (e.g., bilateral temporal lobe atrophy visual score of 0 or 1).

**Table 2:** **Control subjects recruited for the study**.

Subject	Age	MMSE	CDR	Total CERAD	Sex
0001	77	29	0	135	M
0003	58	28	0	117	M
2005	43	28	0	86	M
2006	31	29	0	85	M
2008	32	30	0	85	M

##### Dose administration

A single dose consisted of a 1/2″ ribbon (approximately 0.05 cc) of Compound #11 Ointment (0.5% concentration) topically administered to the inside of the lower eyelid with a syringe applicator. Each dose of Ointment was applied to the study eye of the participant by a trained technician, ophthalmologist, or nurse at the study site.

The participant was asked to keep the dosed eye closed. The dosed eye was covered with an eye cup for 30 min after application of each dose. Each participant in the study received all six doses of fluorescent ligand.

##### Safety

Safety was evaluated by documentation of AEs, by assessment of clinical laboratory findings, by physical examination, including measurement of vital signs, and by ophthalmic examination.

1. Ophthalmic examinations were performed at intervals previously called-out in Protocol NEU02 (see Table A1 in Appendix). Each ophthalmic examination was performed by a Board Certified ophthalmologist who evaluated any signs and symptoms observed vs. the subject’s baseline measures.
2. Ophthalmic exams included assessments of best spectacle corrected visual acuity (BSCVA), uncorrected visual acuity (UCVA), and intraocular pressure (IOP).
3. To assess the possibility that an AE had occurred, subjects were asked at each visit if they had experienced any symptoms since their last examination. Questions were asked in an “open-ended” manner, so as to solicit all inputs from subjects regarding all potential AEs.
4. Physical examinations and clinical laboratory measures were taken before and after each exposure to the test product. In addition, a 7-day post study examination was conducted by the investigator and samples collected for clinical laboratories of both blood and urine. The results were reviewed for any clinically significant changes.

##### Region of interest

**Figure 5:**
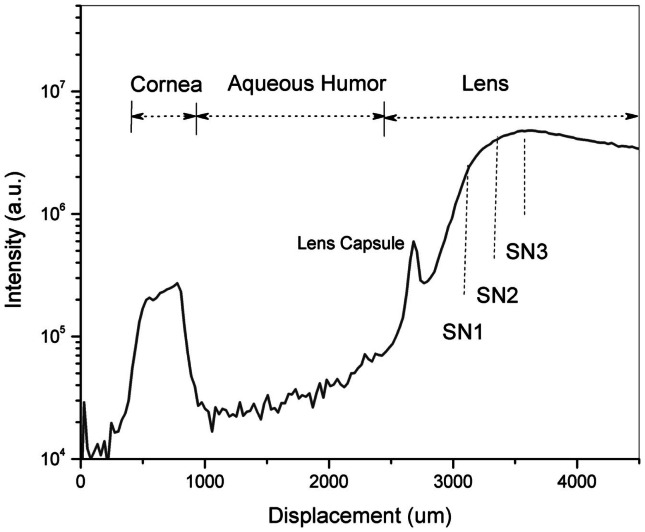
**A *Z*-Scan along the optical axis of the eye of a healthy volunteer to identify the region of interest – the supranucleus (SN) of the lens**. Fluorescence measurements were performed at three different locations designated as SN1, SN2 and SN3.

**Figure 6:**
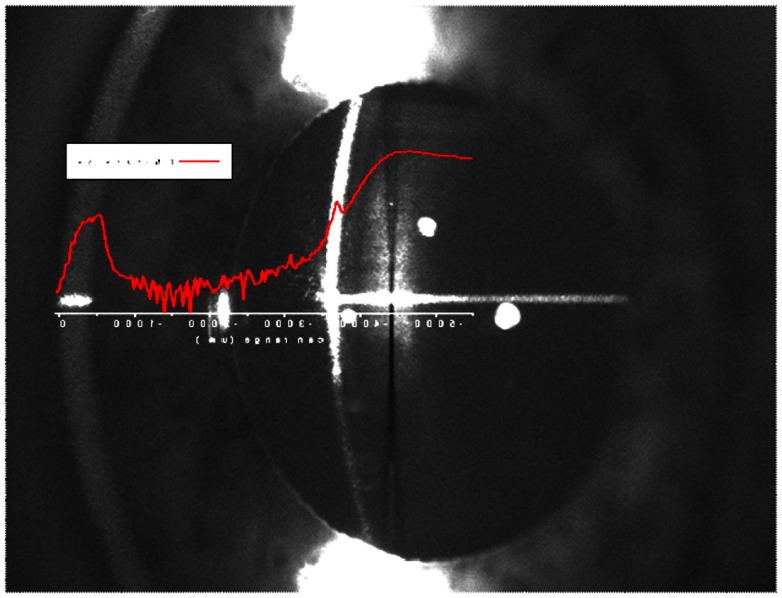
**Overlay of a *Z*-Scan on an oblique image of a healthy volunteer**.

According to the research conducted on post-mortem lenses (Goldstein et al., 2003), Aβ aggregates were reported to develop in the SN of the lens of AD patients. To locate the ROI, i.e., the SN of the lens, an axial scan was performed along the optical axis of the eye by displacing the objective lens with a translation stage (Micronix, Irvine, CA, USA). By exciting natural fluorophores, we indentify the ocular tissues in the eye and hence the desired location in the lens of each individual. Each scan is a two-dimensional plot of photon counts as a function of displacement of the objective lens (in air). Figure 5 is an example of a *Z*-Scan in the eye of a healthy volunteer showing on a logarithmic scale the intensity (photon counts/s) vs. displacement. The intraocular distances can then be determined and the ROI in the SN can be located to perform fluorescence lifetime measurements. In this study, measurements are performed at three different locations in the SN (SN1, SN2, and SN3) to investigate the location of the maximum fluorescent signal indicative of the bound Aβ complex. The distance between each region is about 300 μm and the SN3 region is about 900 μm from the lens capsule. Another illustration of the *Z*-Scan is shown in Figure 6 overlay on an oblique image of the anterior segments of the eye.

Once the location is known, lifetime measurements are initiated over a scanned area (50 μm × 50 μm). To obtain the frequency counts of photons (i-photons/s) with the specific decay lifetime pertaining to the Aβ complex labeled with Compound #11.

##### Measurement sessions

Three different measurement sessions with the SAPPHIRE System were performed: at Baseline, 12 and 24 h point. Each measurement consisted of a *Z*-Scan to determine the ROI followed by fluorescence scanning lifetime measurements at three different locations (three times each) within the SN of the lens. The three locations are noted as SN1, SN2, and SN3 that cover most of the peripheral lens region within 300 μm separation starting from the lens capsule.

After the baseline measurements with the instrument were performed, the ligand was topically administered on the eye of the recruited subjects three times at 2 h (±30 min) intervals. Same procedure was followed at 12 h point (morning session of the next day) and 24 h after the baseline visit.

## Results

### Safety

This initial safety study was designed to assess risks associated with the use of the system in normal subjects (Controls) and Probable AD patients over a period of time that is the same as will be utilized in future trials. Overall, no serious AEs were observed. The SAPPHIRE System, device and the Ophthalmic Ointment, used in this study posed no substantial safety risk. During the course of this study, subjects were administered three applications of Ointment over 4 h period on Visit 2 of the clinical study and, on the next day, administered three additional doses of Ointment over a period of 4 h to the study eye only. The study eye only was illuminated with the device (Class I laser) three times over a 2-day period. Comprehensive ophthalmic examinations were conducted at three time points: before application of the Compound #11 Ointment, after application of each of the treatments with Compound #11 Ointment and employment of the device at Visit 2 and Visit 3. None of the subjects were discontinued from the study for any reason. There were no significant changes in UCVA and BSCVA. Slit lamp biomicroscopy was also performed as a part of the study, along with an ophthalmoscopic examination and the findings were unremarkable as compared between pre-treatment, post-treatment, and follow-up values. No remarkable observations were made by either slit lamp biomicroscopy or from ophthalmoscopy.

Observations of the dilated fundus showed no significant changes during the course of this Safety Study. Dilated fundus examination was completed using an ophthalmoscope to view the inner structures of the eye and included cup to disk ratio, changes in the retinal pigment epithelium and the presence of a nevus, if present, was documented, along with any other abnormalities. After the completion of the trial there were no clinically significant changes that could be attributed to this procedure

### Efficacy

**Figure 7:**
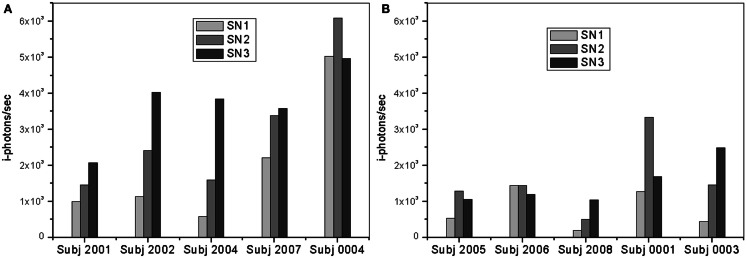
**Plots of *i*-photons/s at 12 h point for (A) AD patients and (B) controls showing all three different locations (SN1, SN2, and SN3)**.

Fluorescence lifetime measurements were performed at three different locations starting from the lens cortex (SN1) into deeper SN region (SN3). As previously stated herein, SN3 is the area that corresponds to the SN of the lens and is about 900 μm from the lens capsule. Figure 7 shows plots of the frequency counts (i-photons/s) of the fluorescence lifetime specific of the bound Compound #11 to Aβ detected for each individual in the two treatment groups of this study.

In both groups, the measurements in the deeper SN region (SN3) show larger signal increase over the other regions (SN1 and SN2). The region is about 900 μm deep from the lens capsule. However, this difference is significantly greater in the “AD Patient” group. In addition, it is interesting to observe that SN3 measurements for Controls are less than half that of AD patient SN3 measurements. Fluorescent intensity measurements in the SN1 and SN2 areas are also higher among AD patients as compared to normal subjects.

**Figure 8:**
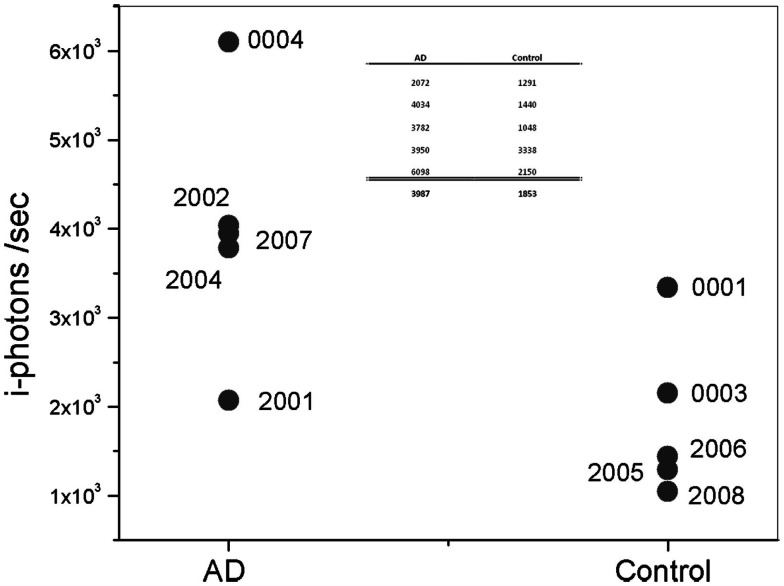
**Plot of maximum *i*-photon/s measured at anyone location**. Table represents the data points for the two groups and their corresponding averages.

The max signal (or frequency count) for each participant, presented in the figure above, is shown more clearly in Figure 8. Overall, Aβ specific frequency counts measured from the AD group are more than twofold higher than corresponding measures in the Control’s group (Table in Figure 8). There is clear separation between the AD patients and Control groups. It is important to mention a few observation points about the results. In particular, Subject 0001 is a normal individual as determined by cognitive testing; however, fluorescent measurements from this individual are higher than from other Control subjects in the cohort, which could be interpreted as a false positive. This patient is a 78-year-old individual with Apolipoprotein E4 variant (ApoE4), known for the genetic risk factor for AD, positive test. There is evidence that Aβ aggregation starts to occur years before symptoms arise. The ApoE4 positive status of this participant increases his risk of developing AD in the future. Patient 2001, on the other hand, had a negative reading with device during which head movements and eye motion interfered with test conduct. It is possible that the movement during the session affected the positioning of the measurements in the eye, and that the exact location in the SN was missed and resulted in low signal.

## Discussion

To the best of our knowledge, this is the first time a clinical study is conducted to detect an exogenous fluorescent signature bound to Aβ in the lens of human being. The clinical study was conducted with no serious AEs related to the use of the fluorescent ligand, Compound #11, or the laser scanning device in the eye. In addition to the safety outcome of this small study, the results obtained from the SAPPHIRE System were encouraging in terms of bioavailability of the ligand in the anterior segment of the eye and the sensitivity of the device to differentiate between the AD and the Control groups. The average signal detected of the AD patients was approximately twice that of Control group. The deeper supranuclear region reveals higher signal as compared to other regions in the SN. The results complement the previous studies by Goldstein et al. (2003) to detect the presence of Aβ in the supranuclear region in lens samples from AD patients. Several techniques were used to analyze Aβ in lens samples including Congo Red staining, western blot, tryptic-digest/mass spectrometry electrospray ionization, and anti-A surface-enhanced laser desorption ionization mass spectrometry, immunohistochemistry, and immunogold electron microscopy that showed a complete or partial circumferential deposit of protein aggregates in the SN. Control lenses from individuals with no evidence of AD, including controls from patients with other neurodegenerative diseases, showed no such deposits.

Moreover, a recent study by Michael et al. (2012) performed on post-mortem lens samples from AD patients located cortical opacities similar to the supranuclear cataracts described by Goldstein et al. (2003). However, the authors were unable to detect Aβ in the lens using amyloidopholic staining methods. The contradictory findings between Goldstein et al. (2003) and Michael et al. (2012) might be due to differences in staining methods of lens samples. These *in vitro* experiments emphasize the importance of *in vivo* studies in the human lens to determine the Aβ presence in AD patients.

The data in this study represent a small population and further investigational clinical studies are underway to include a larger number of participants for statistical analysis and establishment of the SAPPHIRE System as a probable tool to diagnose AD at an early stage.

## Conflict of Interest Statement

The authors declare that the research was conducted in the absence of any commercial or financial relationships that could be construed as a potential conflict of interest.

## Appendix

**Table A1:** **Time and events schedule for the study**.

	Visit 1	Visit 2 (PM)		Visit 3 (AM)		Visit 3 (PM)	Visit 4
	Baseline	SAPPHIRE II	Dose (3×)	SAPPHIRE II	Dose (3×)	SAPPHIRE II	Follow-up exam

	(−0 to −21 days) prior to Visit 2		2 h (±30 min) apart	Next day after Visit 2 before dosing	2 h (±30 min) apart	4 h (±30 min) post-final dose	(7 ± 2 days) after Visit 3
SAPPHIRE II measurements		X		8(+/−1hr)AM		X	
Fluorescent ligand dosing			5(+/−1hr)PM start		X		
Informed consent	X						
Demographic information	X						
Inclusion/exclusion criteria	X						
Cognitive assessments	X						
Medical history	X						
Physical examination	X						X
ECG	X						X
Ophthalmology exam	X					X	X
Vital signs	X	X		X		X	X
Clinical laboratories	X*					X	X*
Adverse events		X	X	X		X	X
Concomitant medications	X	X					X

**Including serum pregnancy test for women of childbearing potential*.
